# Self-efficacy in predicting smoking cessation: A prospective study in Italy

**DOI:** 10.18332/tpc/162942

**Published:** 2023-04-28

**Authors:** Silvano Gallus, Chiara Cresci, Vera Rigamonti, Alessandra Lugo, Vincenzo Bagnardi, Tiziana Fanucchi, Donatello Cirone, Angela Ciaccheri, Salvatore Cardellicchio

**Affiliations:** 1 Department of Environmental Health Sciences, Istituto di Ricerche Farmacologiche Mario Negri IRCCS, Milan, Italy; 2 Anti-smoking center, Careggi University Hospital, Florence, Italy; 3 SOD of Alcohology, Careggi University Hospital, Florence, Italy; 4 Tuscan Regional Alcoholic Center, Careggi University Hospital, Florence, Italy; 5 Department of Statistics and Quantitative Methods, University of Milan-Bicocca, Milan, Italy; 6 ASST Fatebenefratelli Sacco, Milan, Italy

**Keywords:** tobacco smoking, self-efficacy, smoking cessation, smoking cessation service

## Abstract

**INTRODUCTION:**

Predicting the success of smoking cessation might be crucial to guide towards the treatment of smoking dependence in a clinical setting. We analyzed the potential determinants of successful smoking cessation with a specific focus on self-efficacy in predicting quitting smoking.

**METHODS:**

All consecutive smokers (n=478; 224 men and 254 women) attending the Careggi University Hospital Smoking Cessation Service in Florence (Italy) in 2018–2019 provided information on self-efficacy in predicting smoking cessation, using a 1–10 rating scale during their first visit. Patients were followed up for success in quitting smoking at 3, 6 and 12 months, validated through CO exhaled measurement. To evaluate the association between self-efficacy and the probability of success, we estimated multivariable relative risks (RRs) and corresponding 95% confidence intervals (CIs) through log-binomial models for longitudinal data.

**RESULTS:**

Overall, 47.9% of smokers succeeded in their attempt to quit at 3 months, 40.2% at 6 months, and 33.9% at 12 months. Compared to low self-efficacy (rating scale 1–5), the RR of success in quitting smoking was 1.40 (95% CI: 1.06–1.85) for intermediate self-efficacy (scale 6–7) and 1.64 (95% CI: 1.28–2.12) for high self-efficacy (scale 8–10).

**CONCLUSIONS:**

Self-efficacy is an independent determinant of smoking cessation. We recommend to systematically collect self-efficacy, together with other relevant variables, to predict successful smoking cessation. Moreover, strategies to develop and maintain high levels of self-efficacy are essential to increase quit success and improve treatment.

## INTRODUCTION

Worldwide, tobacco smoking causes the death of around 8 million people every year^1^. In Italy, more than 70000 deaths, corresponding to more than 12% of total mortality, are annually attributable to smoking^2^. The large cohort of British male doctors followed for 50 years, between 1951 and 2001, showed that the life expectancy of smokers was 10 years less compared to that of lifelong non-smokers. Also, this study was able to highlight the beneficial effects of smoking cessation estimating the extent of the reduction in mortality when cigarette smoking is stopped at different ages. In particular, it has been shown that subjects who had quit at age 30 or 40 years had practically the same life expectancy as never smokers. Also, quitting at age 60 or 50 years resulted in a gain of about 3 and 6 years of life expectancy, respectively^3^.

In Italy, in 2017–2019, the prevalence of current smokers among adults was around 22% (27% in men and 19% in women) and that of former smokers was approximately 13%^4^. The large majority of Italian former smokers had quit without any help^5^, but today there are pharmacological and psychological supports that greatly increase the rate of success in smoking cessation^6^. In Italy, the National Health Service (NHS) provides these supports to the general population through a number of healthcare providers, mainly pneumologists or psychologists or counsellors and nurses, located in around 300 smoking cessation services (SCSs) of northern, central and southern Italy^7^. In these SCSs, first line medications include varenicline and nicotine replacement therapies (NRT). In addition, individual and group psychological therapies have been shown to increase quit rates^6^.

The possibility to accurately predict the rates of success for a new incoming smoker willing to quit, would allow clinicians of SCSs to best propose a personalized therapeutic course. Accordingly, the international smoking cessation guidelines foresee different therapeutic courses depending by the patient readiness and motivation to quit^8^. For this reason, tools able to predict absolute rates of success for new patients of SCSs would be extremely important to improve their chances of a successful smoking cessation.

Selected sociodemographic and smoking-related characteristics are important determinants of smoking cessation. Thus, advanced age^9^, high level of education, as well as low nicotine dependence^10-12^ and low number of cigarettes smoked per day^11,13^ have all been shown to be directly associated with increased cessation success. Regarding sex/gender, contrasting results have been found in the success of quitting smoking among men and women, with men more likely to maintain long-term abstinence than women^14^. A psychological variable that has been also considered in the evaluation of patient’s process towards smoking cessation is the self-efficacy, that is the awareness of being able to carry out specific activities or to handle situations or aspects of one’s psychological or social functioning to complete a task successfully^15^. In this case it refers to the cognition of patients to be able to quit smoking. Some studies showed that self-efficacy might represent a relatively reliable predictor of smoking cessation^15-17^.

The main objective of this work is to evaluate the main predictors of the successful outcome of smoking cessation attempts in a group of smokers attending the SCS of Careggi Hospital in Florence (Italy) to quit smoking. Since from 2018 the information of smokers’ self-efficacy was added to the items collected at the first visit at the SCS, we analyzed the role of self-efficacy in predicting smoking cessation to verify whether the already existing evidence on the issue was further confirmed in this set of Italian smokers.

## METHODS

We collected data of 576 consecutive current smokers attending for the first time the SCS of Careggi Hospital in Florence (Italy) between January 2018 and December 2019, with the aim of quitting smoking. Overall, 96 subjects who attended only the first visit (without formally starting the course of treatment) and 2 subjects who had a missing value for self-efficacy (i.e. the main outcome of this study) were excluded. Therefore, for the present analysis we considered 478 patients (224 men and 254 women) aged 15–83 years (mean: 54 years). Patients agreed to participate in the study and signed an informed consent form adopted by the Careggi outpatient service.

During the first visit, smokers were asked to provide information on various sociodemographic characteristics, such as sex, age, education level, and marital status. Also data on smoking-related variables were collected and these included smoking intensity (number of cigarettes smoked per day), number of pack-years (number of 20-cigarette packs smoked per day multiplied by the number of years of smoking), number of previous cessation attempts, and the Fagerström test for nicotine dependence (FTND) score. The FTND consists of six questions to establish the degree of physical dependence on nicotine, categorized as ‘low’ (0–4), ‘medium’ (5–7) and ‘high’ (8–10)^18^. Moreover, the healthcare provider measured the concentration (ppm) of carbon monoxide (CO) exhaled. The treating clinician also collected variables related to smoking cessation support and clinical conditions, including waiting time, the physician delivering the treatment, the participation to the motivational group (i.e. a weekly group moderated by professional educators, whose participation is on voluntary basis, proposed in addition to the individual intervention and the agreed therapy), the agreed treatment among pharmacological therapy (i.e. NRT, zyban, varenicline, cytisine), electronic cigarette, or behavioral therapy only (all the patients received a behavioral therapy together with the pharmacological intervention or the electronic cigarette), origin (i.e. whether the subject went to the SCS willingly or if he/she was encouraged by a healthcare professional), and concomitant diseases of the patient. Baseline sociodemographic and smoking characteristics of the 478 patients included in this study are described in Supplementary file Table 1.

**Table 1:** Distribution of patients to evaluate the association between patients’ characteristics and the probability of continuous smoking abstinence at different time evaluations and overall, Careggi Hospital, Florence, Italy, 2018–2019 (N=478)

*Variable*	*n (%)*	*Time*							*Overall*	
		*3 months*		*6 months*		*12 months*			*Univariable analysis^b^*	*Multivariable analysis^c^*
		*% success*	*RR (95% CI)*	*% success*	*RR (95% CI)*	*% success*	*RR (95% CI)*	*p heterogeneity^a^*	*RR (95% CI)*	*RR (95 % CI )*
**All subjects**	478 (100)	47.9	-	40.2	-	33.9	-	-	-	-
**Sex**										
Male (Ref.)	224 (46.9)	44.6	1	38.8	1	32.6	1	-	1	1
Female	254 (53.1)	50.8	1.14 (0.94–1.38)	41.3	1.06 (0.85–1.33)	35.0	1.08 (0.84–1.38)	0.501	1.14 (0.94–1.38)	1.17 (0.98–1.40)
**Age** (years)										
<50 (Ref.)	153 (32.0)	40.5	1	35.3	1	28.8	1	-	1	1
50–60	170 (35.6)	49.4	1.22 (0.95–1.56)	40.0	1.13 (0.85–1.50)	35.9	1.25 (0.91–1.72)	0.891	1.22 (0.96–1.56)	1.22 (0.96–1.54)
>60	155 (32.4)	53.5	**1.32 (1.04–1.68)**	45.2	1.28 (0.97–1.69)	36.8	1.28 (0.93–1.77)	0.980	**1.32 (1.04–1.68)**	**1.35 (1.07–1.71)**
**Fagerström score**										
0–4 (Ref.)	129 (27.0)	49.6	1	41.9	1	35.7	1	-	1	1
5–7	236 (49.4)	51.7	1.04 (0.84–1.29)	42.4	1.01 (0.79–1.30)	37.7	1.06 (0.80–1.40)	0.973	1.04 (0.84–1.29)	1.07 (0.87–1.30)
8–10	113 (23.6)	38.1	0.77 (0.57–1.03)	33.6	0.80 (0.58–1.12)	23.9	0.67 (0.45–1.00)	0.785	0.76 (0.57–1.02)	0.78 (0.58–1.05)
**Self-efficacy score**										
1–5 (Ref.)	139 (29.1)	36.0	1	28.8	1	22.3	1	-	1	1
6–7	152 (31.8)	47.4	**1.32 (1.00–1.74)**	40.1	**1.39 (1.01–1.93)**	34.9	**1.56 (1.07–2.28)**	0.774	**1.33 (1.01–1.76)**	**1.40 (1.06–1.85)**
8–10	187 (39.1)	57.2	**1.59 (1.23–2.05)**	48.7	**1.69 (1.25–2.28)**	41.7	**1.87 (1.31–2.66)**	0.776	**1.60 (1.24–2.07)**	**1.64 (1.28–2.12)**

RR: relative risk. Estimates in bold are statistically significant at 0.05 level.

a The null hypothesis of homogeneity among RRs calculated at different months for each variable of interest was tested using a log-binomial model, including the variable, time, and the interaction between time and variable as covariates. The p-value associated with the interaction parameter was reported.

b RRs were estimated, for each variable of interest, using log-binomial models, including the variable and time as covariates. To account for correlation within subjects, the generalized estimating equations (GEE) method was used. A first-order autoregressive working correlation structure was specified in the model.

c RRs were estimated using a log-binomial model, including all the variables reported in the table and time as covariates. To account for correlation within subjects, the generalized estimating equations (GEE) method was used. A first-order autoregressive working correlation structure was specified in the model.

The self-efficacy in predicting the outcome of cessation attempt was collected during the first visit. This was the self-perception of success of the cessation attempt by the current smokers and was measured on a 1–10 discrete scale ranging from 1, indicating ‘no hope of success’, to 10, indicating ‘confidence of success’.

For each participant, continuous abstinence was assessed through three follow-up visits at 3, 6 and 12 months. During each visit the concentration (ppm) of CO exhaled was recorded and self-reported smoking status and smoking intensity were collected. Self-reported smoking status was assessed at each follow-up and validated through the CO exhaled measurements. Subjects defining themselves as non-smokers were confirmed to be non-smokers if the exhaled CO was ≤6 ppm. Those few subjects defining themselves as non-smokers but with the exhaled CO ≥7 ppm were classified as smokers. Subjects lost at follow-up (165 at 3 months; 204 at 6 months; and 287 at 12 months) were considered to have remained smokers at the corresponding time.

### Statistical analysis

Log-binomial models for longitudinal data were run to evaluate the association between patient’s characteristics and the probability of success of smoking cessation overall and at the different times of evaluation (i.e. 3, 6, and 12 months). To account for correlation within subjects, the generalized estimating equations (GEE) method was used. A firstorder autoregressive working correlation structure was specified in the model. We derived relative risks (RRs) and corresponding 95% confidence intervals (CIs) after adjustment for sex, age, FTND score, and self-efficacy. Self-efficacy, age, and FTND score were considered in approximate tertiles.

Statistical analyses were conducted with the SAS version 9.4 statistical package (SAS Institute, Cary, NC, USA). The statistical significance level was set at a two-tailed p<0.05.

## RESULTS

Of the 478 current smokers, 229 participants (47.9%) were non-smokers at 3 months follow-up (44.6% of men and 50.8% of women), 192 (40.2%) continued abstinence at 6 months (33.8% of men and 41.3% of women) and 162 (33.9%) at 12 months (32.6% of men and 35.0% of women).

**Figure 1:**
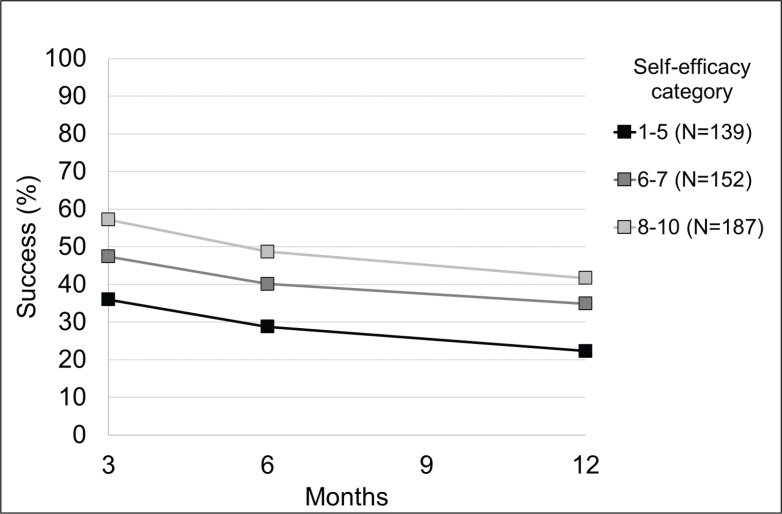
Observed percentages of smoking cessation by time and self-efficacy category, Careggi Hospital, Florence, Italy, 2018–2019 (N=478)

Figure 1 shows the percentage of subjects who successfully quit smoking at various times of evaluation according to self-efficacy score. Smoking cessation was higher among subjects with higher scores of self-efficacy and decreased with increasing time of follow-up.

Table 1 shows the association between patients’ characteristics and the probability of success in smoking cessation at different times of evaluation and overall. Older subjects were more likely to successfully quit smoking compared to younger ones (the multivariable RR for >60 vs <50 years was 1.35; 95% CI: 1.07–1.71). Self-efficacy was directly associated with a success in smoking cessation, the multivariable RR for high (8–10) versus low (1–5) self-efficacy being 1.64 (95% CI: 1.28–2.12). Sex and FTND were not significantly associated with smoking cessation: the multivariable RR for sex (women vs men) was 1.17 (95% CI: 0.98–1.40) and that for high (8–10) vs low (0–4) FTND was 0.78 (95% CI: 0.58–1.05). The adjustment for education level, the delivered treatment, group therapy, and the number of prior quit attempts did not change the multivariable RRs for self-efficacy.

## DISCUSSION

Our study found that self-efficacy represents an independent determinant of smoking cessation in smokers undergoing a cessation program at one SCS in Italy. This result is in broad agreement with those from other studies conducted on the issue. For example, a study based on 600 African American smokers revealed that smokers who successfully quit after six months of follow-up had higher baseline levels of self-efficacy compared to smokers who did not quit smoking^16^. Another study carried out in Switzerland on 115 patients showed that self-efficacy scores in smokers predicted smoking abstinence after 16 months of follow-up^19^. More importantly, a meta-analysis published in 2009, based on 54 prospective studies, suggested that the confidence in the ability to abstain was a consistent predictor of smoking cessation outcomes^20^.

The same meta-analysis, however, showed that the association between self-efficacy and future abstinence is less robust than expected^20^. Accordingly, in our study more than 20% of those with a low baseline level of self-efficacy successfully quit after one year. Moreover, almost 60% of those with a strong confidence to quit failed to be abstinent after 12 months. Although our study did not collect information on motivation and intention to quit, the latter finding might be explained in terms of a relatively limited motivation to quit in these subjects. Indeed, among motivated people, self-efficacy has been shown to play an extremely important role in predicting cessation^21^. It is also possible that those with a strong confidence to quit might refute pharmacological or psychological supports, thus possibly engaging in more high-risk situations^22^.

In our study, among the potential determinants considered, self-efficacy was the strongest predictor of successful smoking cessation. We found a weaker – although statistically significant – direct association between age and smoking cessation. Moreover, in contrast with other studies^23^, we did not find a statistically significant association with smoking cessation for sex or nicotine dependence. A recent study found that having had at least one quit attempt in the prior year was directly associated with quitting self-efficacy^24^. However, even after adjusting the model for the number of prior quit attempts, the main results on the association between self-efficacy and quit success did not substantially change, thus we were not able to demonstrate that the predictive power of self-efficacy is mediated by quit history.

### Limitations

This study has some limitations, including the relatively limited sample size from one single SCS. Moreover, we assessed self-efficacy through one single question before treatment, and not through a validated scale. In addition, the study questionnaire assessed only self-efficacy as psychosocial variable, while other relevant variables, such as attitudes, social norms, motivation and intentions to quit, were not collected. Also, information about the rejected treatment supports was not available. Since these variables are all associated with self-efficacy, the effect of self-efficacy on smoking cessation observed in the study might be partly explained by other unmeasured variables. Finally, a power calculation for the considered sample size was not conducted *a priori*, but all consecutive patients going to the smoking cessation service were included. The study strengths include the prospective study design with a follow-up at 12 months and the validated assessment of smoking status at follow-up, through the measurement of the exhaled CO of all the study participants.

## CONCLUSIONS

In our prospective study conducted in Italy, selfefficacy represents an independent and significant determinant of tobacco abstinence among smokers attempting to quit. We recommend to systematically collect self-efficacy among smokers undergoing a smoking cessation program in a SCS. This variable should be added to sociodemographic and smoking-related characteristics, and motivation to quit, to highly improve prediction models capable of providing a success rate for individual patients attempting to quit smoking^25^. Moreover, healthcare providers should implement strategies aimed at developing and maintaining high levels of self-efficacy and to further motivate smokers with a weak confidence to quit. This can be done by practicing coping strategies for craving and other cessation symptoms, setting achievable goals, and obtaining support from family, friends, or a support group. Increasing self-efficacy and confidence to quit could have major implications from a public health perspective, since there is evidence that these factors are able to increase quit success. Moreover, identifying factors that predict success contributes to improving treatment and moving towards a personalized care for smoking cessation^26^.

## Data Availability

The data supporting this research are available from the authors on reasonable request.

## References

[1] (2019). WHO report on the global tobacco epidemic 2019: offer help to quit tobacco use.

[2] Gallus S, Muttarak R, Martínez-Sánchez JM, Zuccaro P, Colombo P, La Vecchia C (2011). Smoking prevalence and smoking attributable mortality in Italy, 2010. Prev Med.

[3] Doll R, Peto R, Boreham J, Sutherland I (2004). Mortality in relation to smoking: 50 years’ observations on male British doctors. BMJ.

[4] Gallus S, Borroni E, Odone A (2021). The Role of Novel (Tobacco) Products on Tobacco Control in Italy. Int J Environ Res Public Health.

[5] Gallus S, Muttarak R, Franchi M (2013). Why do smokers quit?. Eur J Cancer Prev.

[6] (2020). Linee guida per il trattamento della dipendenza da tabacco.

[7] (2019). Centro Nazionale Dipendenze e Doping, ed. Guida ai servizi territoriali per la cessazione dal fumo di tabacco (aggiornamento maggio 2019).

[8] World Health Organization (2014). Toolkit for delivering the 5A’s and 5R’s brief tobacco interventions in primary care.

[9] Monsó E, Campbell J, Tønnesen P, Gustavsson G, Morera J (2001). Sociodemographic predictors of success in smoking intervention. Tob Control.

[10] Haug S, Meyer C, Ulbricht S (2010). Predictors and moderators of outcome in different brief interventions for smoking cessation in general medical practice. Patient Educ Couns.

[11] Myung SK, Seo HG, Park S (2007). Sociodemographic and Smoking Behavioral Predictors Associated with Smoking Cessation According to Follow-up Periods: A Randomized, Double-blind, Placebo-controlled Trial of Transdermal Nicotine Patches. J Korean Med Sci.

[12] Wu L, He Y, Jiang B (2016). Additional follow-up telephone counselling and initial smoking relapse: a longitudinal, controlled study. BMJ Open.

[13] Jayakrishnan R, Uutela A, Mathew A, Auvinen A, Mathew PS, Sebastian P (2013). Smoking Cessation Intervention in Rural Kerala, India: Findings of a Randomised Controlled Trial. Asian Pac J Cancer Prev.

[14] Smith PH, Bessette AJ, Weinberger AH, Sheffer CE, McKee SA (2016). Sex/gender differences in smoking cessation: A review. Prev Med.

[15] Bandura A (1986). Social foundations of thought and action: a social cognitive theory.

[16] Boardman T, Catley D, Mayo MS, Ahluwalia JS (2005). Self-efficacy and motivation to quit during participation in a smoking cessation program. Int J Behav Med.

[17] Etter JF, Bergman MM, Humair JP, Perneger TV (2000). Development and validation of a scale measuring self-efficacy of current and former smokers. Addiction.

[18] Heatherton TF, Kozlowski LT, Frecker RC, Fagerström KO (1991). The Fagerström Test for Nicotine Dependence: a revision of the Fagerström Tolerance Questionnaire. Br J Addict.

[19] Borland R, Owen N, Hill D, Schofield P (1991). Predicting attempts and sustained cessation of smoking after the introduction of workplace smoking bans. Health Psychol.

[20] Gwaltney CJ, Metrik J, Kahler CW, Shiffman S (2009). Self-Efficacy and Smoking Cessation: A Meta-Analysis. Psychol Addict Behav.

[21] Lee HS, Catley D, Harris KJ (2014). Improving Understanding of the Quitting Process: Psychological Predictors of Quit Attempts versus Smoking Cessation Maintenance among College Students. Subst Use Misuse.

[22] Staring ABP, Breteler MHM (2004). Decline in smoking cessation rate associated with high self-efficacy scores. Prev Med.

[23] Gram IT, Antypas K, Wangberg SC, Lochen ML, Larbi D (2022). Factors associated with predictors of smoking cessation from a Norwegian internet-based smoking cessation intervention study. Tob Prev Cessation.

[24] Al Thani M, Leventakou V, Sofroniou A (2022). Factors associated with baseline smoking self-efficacy among male Qatari residents enrolled in a quit smoking study. PLoS One.

[25] Lai CC, Huang WH, Chang BCC, Hwang LC (2021). Development of Machine Learning Models for Prediction of Smoking Cessation Outcome. Int J Environ Res Public Health.

[26] Coughlin LN, Tegge AN, Sheffer CE, Bickel WK (2020). A Machine-Learning Approach to Predicting Smoking Cessation Treatment Outcomes. Nicotine Tob Res.

