# Supplementary material for: Self-efficacy in predicting smoking cessation: A prospective study in Italy
Source: Tob Prev Cessat. 2023 Apr 28;9:15. doi: 10.18332/tpc/162942 (PMC10141785; doi:10.18332/tpc/162942)
Supplement: Supplementary file 1 [file TPC-9-15-s1.pdf]

Supplementary Table 1. Distribution of 478 current smokers according to selected socio-demographic features and baseline smoking characteristics. Careggi Hospital, Florence, Italy, 2018-2019.

Variable	Levels	N	%
**All subjects**	-	478	100.0
Socio-demographic features			
**Sex**	Men	224	46.9
	Women	254	53.1
**Age (years)**	<50	153	32.0
	50-60	170	35.6
	>60	155	32.4
	Mean (SD)	53.8 (12.9)	
**Level of education**	None or elementary school	34	7.1
	Middle school	168	35.2
	High school	193	40.4
	University	83	17.4
**Marital status**	Single	99	20.7
	Married or cohabitant	295	61.7
	Divorced or separated	64	13.4
	Widowed	20	4.2
**Referral**	Self-referred	17	3.6
	General practitioner	294	61.6
	Specialist physician	95	19.9
	Staff of the Careggi hospital	17	3.6
	Other	54	11.3
Baseline smoking characteristics			
**Fagerström test**	0-4	129	27.0
	5-7	236	49.4
	8-10	113	23.6
	Mean (SD)	5.8 (2.3)	
**Smoking intensity (cigs/day)**	<15	122	25.5
	15-24	228	47.7
	≥25	128	26.8
	Mean (SD)	19.7 (9.9)	
**Pack-years**	Mean (SD)	35.3 (19.8)	

**Exhaled CO (ppm)**	<9	116	24.3
	10-19	196	41.1
	≥20	165	34.6
	Mean (SD)	17.0 (10.5)	
**Previous quit attempts (n)**	0	118	24.7
	1	192	40.2
	2	89	18.6
	≥3	79	16.5

CO: carbon monoxide; ppm: parts per million; SD: standard deviation.

© 2023 Gallus S. et al.
